# The Current Progresses in the Genes and Networks Regulating Cotton Plant Architecture

**DOI:** 10.3389/fpls.2022.882583

**Published:** 2022-06-09

**Authors:** Xianzhong Huang, Hui Liu, Bin Ma

**Affiliations:** ^1^Center for Crop Biotechnology, College of Agriculture, Anhui Science and Technology University, Chuzhou, China; ^2^State Key laboratory of Plant Cell and Chromosome Engineering, Institute of Genetics and Developmental Biology, Chinese Academy of Sciences, Beijing, China; ^3^Plant Genomics Laboratory, College of Life Sciences, Shihezi University, Shihezi, China

**Keywords:** cotton, plant architecture, genetic improvement, florigen and antiflorigen, flowering transition

## Abstract

Cotton is the most important source of natural fiber in the world as well as a key source of edible oil. The plant architecture and flowering time in cotton are crucial factors affecting cotton yield and the efficiency of mechanized harvest. In the model plant arabidopsis, the functions of genes related to plant height, inflorescence structure, and flowering time have been well studied. In the model crops, such as tomato and rice, the similar genetic explorations have greatly strengthened the economic benefits of these crops. Plants of the *Gossypium* genus have the characteristics of perennials with indeterminate growth and the cultivated allotetraploid cottons, *G. hirsutum* (Upland cotton), and *G. barbadense* (Sea-island cotton), have complex branching patterns. In this paper, we review the current progresses in the identification of genes affecting cotton architecture and flowering time in the cotton genome and the elucidation of their functional mechanisms associated with branching patterns, branching angle, fruit branch length, and plant height. This review focuses on the following aspects: (i) plant hormone signal transduction pathway; (ii) identification of cotton plant architecture QTLs and PEBP gene family members; (iii) functions of *FT*/*SFT* and *SP* genes; (iv) florigen and anti-florigen systems. We highlight areas that require further research, and should lay the groundwork for the targeted bioengineering of improved cotton cultivars with flowering times, plant architecture, growth habits and yields better suited for modern, mechanized cultivation.

## Introduction

The core aspects of the aerial architecture of plants are determined by the overall plant height, stem growth habits, and branching pattern, while the peripheral structural features reflect the size, shape, quantity, and relative arrangements of the leaves and flowers. The architecture of plant is species-specific, reflecting a strict genetic control. However, genes with an important role in determining plant structure can be influenced by many exogenous factors, such as light, temperature, humidity, and plant nutritional status (Reinhardt and Kuhlemeier, 2002; Wang et al., 2018). In flowering plants, the shoot apical meristem (SAM) and root apical meristem contains a large number of pluripotent stem cells that are maintained by continuous or periodic division and from which, the different cell lineages are formed through differentiation and continuously form new tissues and organs, such as leaves, stems, branches, and flower (Bowman and Eshed, 2000; Barton, 2010).

In agronomically important crops, plant architecture has a strong influence on the methods of crop cultivation and management as well as the design and efficiency of mechanized plant harvest. Changing the flowering time can enhance the ecological adaptability of plants, and changing the plant architecture can optimize the planting mode of crops. Therefore, flowering time and plant architecture traits are important goals of crop genetic improvement. Lang et al. (1977) first described the balance between positive flowering and negative flowering signals through grafting experiment in tomato and tobacco, i.e., the later identified florigen and antiflorigen (Lang et al., 1977; McGarry and Ayre, 2012a; Eshed and Lippman, 2019). Florigen and gibberellin systems control flowering time and plant architecture. The occurrences of natural mutations in these systems and their exploitation or bioengineering efforts have led to improved crop traits. For example, in the green revolution of the 1960s wheat varieties with shorter and sturdy stalks were selected for in breeding programs, so that crops could better resist lodging and weather hazards while maintaining a higher yield (Peng et al., 1999). By studying the tomato *SELF-PRUNING* mutant, scientists discovered the first antiflorigen molecule SP (Pnueli et al., 1998). In the model plant species, *Arabidopsis thaliana*, research into the gene functions related to plant height, flowering time and the formation of branch and inflorescence structures have been studied extensively. Research on crops of agronomical importance, such as tomato and rice, and the subsequent identification of gene targets for genetic breeding programs has greatly enhanced the economic benefits of these crops (Wang et al., 2018).

**FIGURE 1:**
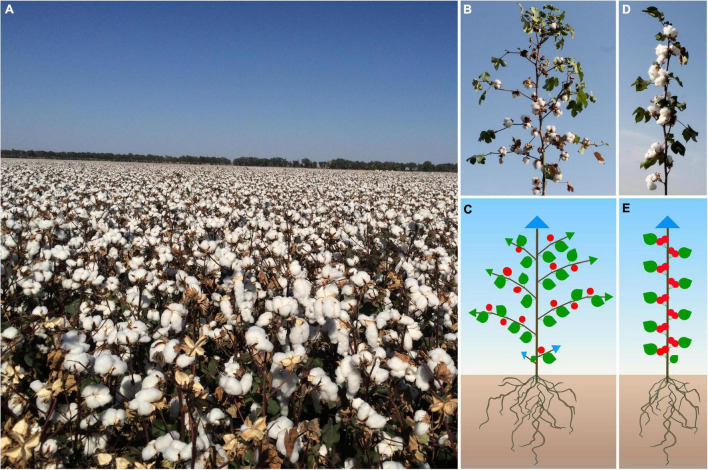
The cultivation feature and plant architecture of cotton. **(A)** The planting pattern of short-dense-early cotton in Xinjiang Uygur Autonomous Region, China. Phenotype of normal fruit branch upland cotton **(B)** and its schematic diagram **(C)**. Phenotype of short fruit branch upland cotton **(D)** and its schematic diagram **(E)**. Blue triangles indicate monopodial shoot apical meristem; green triangles represent sympodial shoot meristem; red balls represent determinate floral buds; green peach-like shapes indicate leaves.

Cotton originated in tropical and subtropical regions, where it showed indeterminate growth and perennial growth habit, and cultivated cotton gradually lose its sensitivity to photoperiod, mainly annual. Cotton plant architecture and flowering time are the key factors affecting cotton yield and the efficiency of mechanization cultivation. Because, compared with the potential of increasing yield per plant, increasing the planting density has greater potential to increase the yield. In Xinjiang Uygur Autonomous Region, China, the main cultivation feature of cotton is dwarf, dense and early (Figure 1A), which has made great achievements in cotton production in Xinjiang. The planting density in this area is generally 180,000–220,000 plants per hectare, making it the largest and highest yield cotton planting area in China (Si et al., 2018; Zhan et al., 2021; Figure 1A). Cotton plant architecture is determined by many factors such as branching pattern, inflorescence structure and plant height, and its branching architecture is different from Solanaceae plants such as tomato (Park et al., 2014a; Moraes et al., 2019). The main stem of cotton displays monopodial branching, which maintains the characteristics of indeterminate growth and produces vegetative growth indefinitely. There are two kinds of branches in cotton. The basal vegetative branches are monopodial and show an indeterminate growth habit. The distal fruit branches show a sympodial inflorescence and each sympodial unit is composed of internodes, a terminal boll with flowers, an opposite leaf and an axillary bud that produces the next sympodial unit, thus forming a pyramidal plant structure (Figures 1B,C; Gore, 1935; McGarry et al., 2016; Si et al., 2018). In this paper, we will review recent progress in the identification and characterization of genes and quantitative trait loci (QTL) regulating cotton branching pattern, branching angle, fruit branch length, plant height and flowering time (Table 1), as well as their application and/or potential in breeding programs to obtain beneficial agronomical trait in cotton. A greater understanding of the genetic regulatory network of these progresses will lay the foundation for future targeted bioengineering of improved cotton cultivars for higher yields and optimized cultivation and harvesting.

**TABLE 1:** The identified genes and QTLs related to cotton plant architecture.

Gene	Description	Functions	References
*GhFT1*	*FT* ortholog	Flowering, lateral shoots	Guo et al., 2015
*GhSFT*	*SFT* ortholog	Flowering and determinate growth	McGarry et al., 2016
*GhTFL1*	*TFL1* ortholog	Delay flowering	Prewitt et al., 2018
*GhSP*	*SP* ortholog	Branching pattern	McGarry et al., 2016; Si et al., 2018
*GbSP*	*SP* ortholog	Nulliplex fruit branch	Si et al., 2018
*GhNB*	*TFL1* ortholog	Nulliplex-branch	Chen et al., 2019
*GoCEN-Dt*	*CEN* ortholog	Cluster fruiting and early maturity	Liu et al., 2018
*GhFD1/2*	FD orthologs	Flowering	Liu et al., 2021
*GhGRF14*, *GhGRF3/6/9/15*	General regulatory factor	Flowering	Sang et al., 2021
*GhBRC1*	*TEOSINTE BRANCHED1*	Secondary shoot formation	Sun et al., 2021
*GhTIE1*	TCP interactor containing EAR motif protein 1	Shoot branching	Diao et al., 2019
*GhDREB1B*	a dehydration-responsive element-binding transcription factor	Plant height and branch length, and reduced branch angle	Ji et al., 2021
*GbDWARF14*	An unconventional Strigolactones receptor	Branch development	Wang et al., 2019
*GhNCED1*	Nine-cis epoxycarotenoid dioxygenase	Plant height	Pei et al., 2021
*GhPAS1*	*PAGODA1 SUPPRESSOR 1*	Plant height	Wu et al., 2021
*Gh_D03G0922*	*MADS-box*	QTL for plant height	Su et al., 2018
Ghir_D02G017510, Ghir_D02G017600		Candidate gene influencing plant height and fruit spur branch number (FSBN). qD02-FSBN-1,	Wen et al., 2019
*Ghir_A12G026570*	Pectin lyase-like superfamily	qA12-FSBN-2	Wen et al., 2019

## The Regulatory Roles of Plant Hormone Signaling Pathways in the Formation of Cotton Plant Architecture

Plant hormones play an important role in regulating meristem activity, which has a profound influence on the establishment of plant architecture (Wang et al., 2018). The introduction of dwarfing traits into wheat and rice during the “Green Revolution” is now understood to have been achieved through the genetic alteration in gibberellin metabolism and action (Peng et al., 1999; Sasaki et al., 2002). In *Arabidopsis thaliana*, the role of hormones and their signaling pathways in plant development has been studied intensively (Ongaro and Leyser, 2008). However, there has been comparatively little systematic research into the role of plant hormones and their underlying molecular mechanisms in the regulation of cotton architecture and flowering. Nevertheless, some progress has been made through the identification and functional analysis of cotton orthologous genes important for hormonal signaling and their targets in mode plant systems.

Signals derived from several factors, including hormones, nutrients, and environmental factors, cooperatively regulate the growth of buds and their related signal pathways converge on BRANCHED1 (BRC1), which ultimately determines whether axillary buds are dormant or growing (González-Grandío et al., 2013; Wang et al., 2018). *BRC1* gene encodes a TCP transcription factor, which is specifically expressed in axillary buds and participates in the inhibition of bud growth and branching (Hubbard et al., 2002). Maize TB1 is an ortholog of BRC1 in *A. thaliana*, which has been studied intensely for its regulatory role in branching patterns (Hubbard et al., 2002; Wang et al., 2018).

*GhBRC1* is highly expressed in the axillary bud tissue of cotton with nulliplex-branch (no fruit branching) phenotypes, and is induced by various hormones (Sun et al., 2021). The overexpression of *GhBRC1* in *A. thaliana* resulted in a significant reduction in the rosette-leaf branch number. In addition, transformation of *A. thaliana* mutant, *brc1*, rescued its high branch number phenotype (Sun et al., 2021). Silencing this gene in cotton using virus-induced gene silencing (VIGS) caused early flowering and formed a multi-branched phenotype, suggesting that similar to *AtBRC1*, *GhBRC1* is a key gene exerting negative control over the branch growth and development (Sun et al., 2021).

TIE1 (TCP interactor containing EAR motif protein 1), a transcriptional repressor, interacts with and represses BRC1 activity, thus acting as a positive regulator of branching (Tao et al., 2013). The ectopic overexpression of *GhTIE1* in *A. thaliana* led to a more highly branched phenotype, whereas silencing of *GhTIE1* using VIGS technology decreased branching number in cotton, suggesting that *GhTIE1* positively regulates shoot branching (Diao et al., 2019). The results showed that TIE1 exerts a conserved regulatory function over branching in arabidopsis and cotton, through modulations of the activities of TIE1 and TCP proteins, including BRC1 (Diao et al., 2019).

As one of the largest families of transcription factors in plants, NACs (NAM, ATAF1/F2 and CUC2) plays important roles in the formation and maintenance of different meristems and the development of bud branches. GhCUC2 interacts with GhBRC1 to negatively regulate cotton branching, while miR164 has the opposite effect and inhibits the function of GhCUC2 by cleavage of its mRNA. GhBRC1 can directly bind to the promoter region of *NCED1* (*Nine-Cis Epoxycarotenoid Dioxygenase*) and activate the transcription of this gene, resulting in local ABA accumulation with the suppression of axillary bud and lateral branch formation. The miR164-GhCUC2-GhBRC1-GhNCED1 module is therefore an important regulatory axis in lateral branch development in arabidopsis and cotton (Zhan et al., 2021). The expression levels of the cotton *NCED* homologous genes in dwarf accessions were higher than that in taller ones. Silencing of *GhNCED1* using VIGS increased plant height and internode numbers (Pei et al., 2021), suggesting that *GhNECD1* negatively regulate plant height.

Strigolactone (SL) is considered an important plant hormone in the regulation of branch development. SL promotes the expression of *TB1*/*BRC1* and thereby participates in the inhibition of axillary bud development and plant branch formation (Gomez-Roldan et al., 2008). DWARF4 (D14) is a α/β hydrolase in the SL signaling and a non-canonical receptor of SL, forms a covalent interaction with the SL hydrolysis product (Yao et al., 2016). The ortholog of *D14* in sea-island cotton, *GbD14*, was found to be highly expressed in axillary buds, and the ectopic expression of *GbD14* in *A. thaliana* could complement the multi-branched phenotype of the *Atd14* mutant (Chevalier et al., 2014), confirming that *GbD14* was involved in the regulation of cotton branching (Wang et al., 2019). *GhPAGODA1* (*PAG1*) encodes a cytochrome P450 that catabolizes active brassinosteroid (BRs) *via* C-26 hydroxylation (Yang et al., 2014). *GhPAGODA1 SUPPRESSOR 1* encodes a bHLH transcription factor of cotton, and *GhPAS1* overexpression in arabidopsis could partially rescue the dwarf phenotype of *Atpag1* mutant; while *GhPAS1* silencing using VIGS inhibited plant growth and altered plant architecture in cotton (Wu et al., 2021). This result suggested that GhPAS1 regulates cotton development and architecture *via* mediating BR signaling.

In summary, from recent research into the hormonal regulation of branching in *A. thaliana* and rice, several important genes with key roles in this process have been characterized and their functional orthologs in cotton have been identified. However, the current research in cotton is still quite superficial and the number of key genes identified is still very limited. *AiSheng*, a natural dwarf mutant of cotton, has shorter fruit branches and presents at a more acute angle to the stems. Gene cloning revealed that the gene located on chromosome D12 and contained a tandem duplication of about 13.5 kb, which led to the doubling of the copy number of *Dehydration Responsive Element Binding factor* (*GhDREB1B*), with a significant increase in gene expression, resulting in phenotypic changes (Ji et al., 2021). RNA-seq analysis showed that the relative up-regulation of *GhDREB1B* down-regulated many genes of the auxin signaling pathway, but up-regulated many genes of the ethylene and BR signaling pathways, which might comprehensively lead to a more compact cotton plant architecture (lower plant height, shorter branches, and smaller included angle to the stem). This study showed that *GhDREB1B* was an important gene controlling cotton plant architecture, through its effects on hormones signal pathways (Ji et al., 2021). The present findings enrich the regulatory network of cotton architecture and provide candidate genes for precise control of cotton form.

## Identified Quantitative Trait Locus Related to Cotton Architecture

Genetic maps have been used in cotton, and some new technologies developed with the sequencing of cotton genome have been used to fine locate QTLs related to cotton structural traits (Song and Zhang, 2009; Huang et al., 2017; Su et al., 2018; Wen et al., 2019; Grover et al., 2020; Wang et al., 2022).

Song and Zhang (2009) identified 26 single QTLs with seven plant architectural traits using the interspecific hybrid BC1 population of *G. hirsutum* and *G. barbadense*. Huang et al. (2017) identified 160 candidate QTLs associated with 16 agronomic traits, including QTLs for plant height, fruit branch length using CottonSNP63K array and 503 Chinese upland cotton materials.

Using 355 upland cotton accessions, a genome-wide association study (GWAS) identified one gene, *Gh_ D03g0922*, affecting plant height (Table 1; Su et al., 2018). This gene encodes a protein containing MADS box domain, which was up-regulated in the apical buds and young leaves of short and compact cotton varieties (Su et al., 2018). Also, GWAS have identified five QTLs controlling plant height and six QTLs for fruit spur branch number using 121 cotton varieties, including 100 brown-fiber cotton and 21 white-fiber accessions (Wen et al., 2019). A restricted two-stage multilocus GWAS (RTM-GWAS) have identified 68 gene loci related to plant height, 34 to fruit branch angle and 55 to fruit branch length in 315 natural populations of upland cotton (Wang et al., 2022).

Ten plant architecture traits were evaluated, which include plant height, fruiting branch length, branch angle, and stem pubescence using an intraspecific cross population within *G. hirsutum* (Grover et al., 2020). 120 QTLs related to phenotypic changes under domestication have been identified. Many QTLs are both clustered and environmentally labile (Grover et al., 2020).

Although QTL regions contain many genes, the identified QTLs related to cotton plant architecture provide targets for further mining candidate genes and key insights for in-depth understanding of the genetic basis of cotton plant architecture.

## Knowledge on the Phosphatidylethanolamine Binding Proteins Gene Family of Cotton

Phosphatidylethanolamine binding proteins (PEBPs) are also referred to as CETS from [CENTRORADIALIS (CEN)/TERMINAL FLOWER1 (TFL1)/SELF-PRUNING (SP)] proteins (McGarry and Ayre, 2012a; Lifschitz et al., 2014). The PEBP of higher plants contain three subfamilies, including the MOTHER OF FT AND TFL1 (MFT)-like proteins, the FLOWERING LOCUS T (FT)-like proteins and the TFL1-like proteins (Karlgren et al., 2011). In the *TFL1*-like clade, there are multiple genes including *TFL1*, *BROTHER OF FT*, and *SP* genes. Of these, FT protein is the florigen of plant, whereas TFL1, SP, and CEN proteins are antiflorigen components. They constitute the florigen and antiflorigen system of plants, which is essential to maintain the determinate and indeterminate growth of meristem (Lifschitz et al., 2014; Eshed and Lippman, 2019). Therefore, these genes are the key regulatory factors of plant architecture, controlling the transition to flowering and the meristem fate.

In recent years, the successful sequencing and sequence update of cotton genome has led to the identification of PEBP gene members by several research groups (Table 1; Hu et al., 2019; Huang et al., 2020). McGarry et al. (2016) and our genome-wide identification results showed that there were eight *PEBP* genes in diploid cotton (*G. raimondii* and *G. arborum*), and eight pairs of *PEBP* genes in tetraploid cottons (*G. hirsutum* and *G. barbadense*), of which A_t_ and D_t_ contained eight loci, respectively (McGarry et al., 2016; Si et al., 2018). In the *G. hirsutum* genome, there are two homoeologous *MFT* gene pairs (*GhMFT-L1A*/*L1D*, *GhMFT-L2A*/*L2D*) and one homoeologous *FT* gene pair (*GhFT-A* and *GhFT-D*, or named [*GhSFT-A* and *GhSFT-D* by McGarry et al. (2016)], two homoeologous pairs of *TFL1* genes (*GhTFL1-1A*/*1D*, *GhTFL1-2A*/*2D*), one homoeologous pairs of *SP* genes (*GhSP-A*/*D*) and two homoeologous pairs of *BFT* genes (*GhBFT-1A/1D*, *GhBFT-2A/2D*). Most *PEBP* genes have similar intron and exon structures, and all PEBP proteins contain very conservative “D-P-D-x-P” and “G-x-H-R” motifs and key amino acids to distinguish FT and TFL1 proteins (Ahn et al., 2006). These results indicate that more *PEBP* gene members have been produced during the evolution of the cotton genome, and also imply the complexity of *PEBP* gene functions in cotton.

## Functions of Flowering Locus T/Single Flower Truss Genes in Cotton

Florigen, encoded by *FT*, mainly maintains determinate growth and promotes flowering transition, which is not only functionally conserved in evolution, but also displays pleiotropic trait (Moraes et al., 2019; Jin et al., 2021). Plants perceive changes in the photoperiod in leaves and promote the expression of *FT* in leaf companion cells to synthesize the FT protein. At low temperatures, FT can bind to negatively charged phosphatidylglycerols in the cell membrane of companion cells, thus reducing the level of soluble FT in sieve tubes which, limits the long-distance transport of FT protein to the apical meristem and delays the transition to flowering (Susila et al., 2021). This study reveals the interactions between FT and phosphatidylglycerol and its molecular mechanism of jointly regulating plant flowering process, which provides a new research idea for the mechanism of plants adjusting flowering process in response to seasonal temperature changes. At more favorable temperatures, FT can be more easily transported to the shoot apex, where it forms a complex with 14-3-3 proteins (Susila et al., 2021). After transport to the nucleus, the complex binds with the bZIP transcription factor, FD, to form a flowering activation complex (FAC), which induces flowering through the activation of downstream flower identity genes, such as *SUPPRESSOR OF OVEREXPRESSION OF CONSTANS 1* (*SOC1*) and *APETALA1* (*AP1*) (Abe et al., 2005; Wigge et al., 2005; Taoka et al., 2011).

Some studies have revealed that *FT* orthologs are functionally conserved in different plant species. The ectopic overexpression of *AtFT* in wild and cultivated cotton using the virus-mediated transformation system enhanced the determinate growth habit of cotton aerial parts by the decoupling of flowering from photoperiod regulation to produce early flowering and accelerated ripening, with a dense growth of fruit branches on the main stem (McGarry and Ayre, 2012b). However, it did not affect the structural development of the cotton flower organs formed (McGarry et al., 2013). Similarly, the upregulation of *GhFT/SFT* expression in cotton mediated by a disarmed *Cotton leaf crumple virus* caused cotton flowering to separate from photoperiod leading to premature flowering (McGarry et al., 2016; McGarry and Ayre, 2021). However, the indeterminate growth habit of the main branches did not change, although some fruit branches became shorter or grew directly on the main stem. Conversely, the silencing of *GhFT/SFT* mediated by the tobacco rattle virus led to delayed cotton maturation and the display of more characteristics of indeterminate growth, such as larger main stem leaves and longer petioles (McGarry et al., 2016). Furthermore, Prewitt et al. (2018) reported that *GhSFT* rescued *Atft-10* mutant, having similar numbers of leaves and flowering times with wild type. These studies show that the *FT* gene affects the plant architecture of cotton and that the judicious manipulation of *FT* and related genes could weaken perennial characteristics to improve domesticated strains. Our study indicated that overexpression of *GhFT* in *A. thaliana* promoted flowering and partially complemented the late flowering phenotype of the *Atft-10* mutant, suggesting that *GhFT* is similar to the FTs of other plant species in the regulation process of controlling flowering (Guo et al., 2015; Cai et al., 2017; Sang et al., 2019). Previous studies have shown that the FT protein, as a general florigen, regulates multifaceted growth and development processes of plants (Shalit et al., 2009). We also showed that ectopic overexpression of *GhFT* in tobacco not only causes early flowering, but also promotes the overgrowth of lateral branches, leaf development, flower abscission. Molecular analyses showed that ectopic expression of *GhFT* in tobacco might affect the distribution of endogenous FT, affecting the plant morphogenesis (Li et al., 2015).

The FAC model reveals the functional conservation of florigen complex, because the three proteins constituting FAC are very conserved in seed plants (Karlgren et al., 2011; Taoka et al., 2011). Through genome-wide identification and analysis, we found that there were five *FD*s and 31 *14-3-3* homoeologous gene pairs in *G. hirsutum* (Liu et al., 2021; Sang et al., 2021). GhFT–Gh14-3-3–GhFD1/2 complexes can activate the expression of *GhAP1*,the homologous flower identity gene in cotton, thus promoting the flowering. In contrast, GhFT–Gh14-3-3–GhFD3/4/5 complexes in roots activate the expression of *AUXIN RESPONSE FACTOR 19*, thereby promoting lateral root development (Liu et al., 2021). The function of FAC in cotton is also influenced by its component 14-3-3 protein, which can either promote or inhibit flowering by regulating the expression of *GhAP1* and *GhSOC1* (Sang et al., 2021). These studies show that although there is only one FT/SFT protein in polyploid upland cotton, it possesses diverse FD and 14-3-3 proteins available allowing the formation of diversified FACs to regulate different development processes.

## Cotton Terminal Flower1/Self-Pruning/Centroradialis Genes Regulate the Development of Fruit Branches

TFL1/SP/CEN proteins are also homologous to FT, but have an opposite function. ***TFL1***/***SP***/***CEN*** mainly promotes indeterminate activity in meristem to maintain vegetative growth and inhibit the transition to flowering. Loss-of-function mutations in *AtTFL1* and *AmCEN* decrease plant height with the onset of precociously flowering (Bradley et al., 1996, 1997). The tomato *sp* mutant is particularly short, dense and compact with restricted growth in the stems and branches which, avoids the necessity for pruning, enabling the tomato to be planted on a large scale (Pnueli et al., 1998). The mutation of the *DETERMINATE STEM* gene, a soybean ortholog of the arabidopsis *TFL1*, leads to a shorter and more compact plant, with a concentrated fruit setting (Tian et al., 2010). Therefore, TFL1/SP proteins in plants constitute the antiflorigen system and play a decisive role in establishing plant architecture. Crop *TFL1/SP* mutations are widely used in agriculture due to their better adaptation to dense planting regimes and mechanized harvesting with improvements in yield and reduced planting costs (Eshed and Lippman, 2019).

Although most of the modern cottons cultivated today are daily neutral and annual crops, the perennial habit of indeterminate growth of their ancestral varieties still persists to some degree. Some cotton mutants have been characterized as deficient in the cotton orthologous of tomato *SELF-PRUNING* (*GoSP*) and these display axillary flowers on short or nulliplex fruit branches (Chen et al., 2019). This results in a more compact architecture, allowing high-density planting to increase cotton yield. There is also a cluster boll mutation in upland cotton, in which one or more cluster flowers with slender pedicels form on the main stem (Figures 1D,E; Pathak and Singh, 1975). Almost all the long-staple cotton varieties cultivated and planted in Xinjiang display short fruiting branches. This kind of short fruit branch character makes it possible to increase the planting density and greatly increase the cotton yield. Therefore, short branch character is an ideal plant type for cotton breeding. This phenotype was found simultaneously by us and two other independent research groups to be due to mutations in a cotton ortholog of *TFL1/SP/CEN*. Although all three research groups identified the same gene, it was given different names by each (Liu et al., 2018; Si et al., 2018; Chen et al., 2019). Because the amino acid sequence deduced from this gene has the highest similarity with the tomato SP protein, this paper describes it as the cotton *SP* gene (McGarry et al., 2016; Si et al., 2018). Chen et al. (2019) reported four types of *Ghsp* mutations in upland cotton and that *GhSP* was mainly expressed in roots and shoot apexes. Overexpression of the *GhSP* gene in cotton inhibited flower bud growth, indicating that *GhSP* plays a key role in regulating flower bud differentiation and determinate growth (Chen et al., 2019). *In situ* hybridization revealed that *GhSP* was preferentially expressed in the meristems of cotton main stems and axillary buds (Liu et al., 2018). Overexpression of *GhSP* inhibited the transition from vegetative to reproductive branch formation (Liu et al., 2018; Si et al., 2018). Conversely, the silencing of *GhSP* resulted in early flowering and determinate growth, with the production of terminal flowers on the main stem and shorter lateral branches (McGarry et al., 2016; Liu et al., 2018). RNA-seq analysis showed that this phenotypic alteration generally occurred with the upregulation of a *MADS*-box gene (Liu et al., 2018). It was found that all varieties of sea-island cotton in Xinjiang with *sp* mutation displayed early maturity, indicating that they are better adapted for cultivation at high latitudes with short frost-free periods, indicating that the *sp* mutation had been strongly selected for early maturity breeding program (Liu et al., 2018; Si et al., 2018).

Similarly, our research revealed that the mutation of *Gbsp*^113^*^Ser^* in sea-island cotton produces nulliplex-branches, while the mutation of *Ghsp*^73^*^Asn^* in upland cotton produces cluster-bolls (Si et al., 2018). However, in both mutations, the apical meristem maintains indeterminate growth. In addition, when *Ghsp*^73^*^Asn^* or *Gbsp*^113^*^Ser^* gene was overexpressed in arabidopsis *tfl1-1* mutant, the dwarfed, early flowering and multi-branched phenotypes of the *Attfl1-1* mutant were not changed (Si et al., 2018). *GhSP* silencing terminates the indeterminate growth of cotton, leading to the termination of the monopodial main stem, thus leading to early flowering and early maturity. Subcellular localization analysis showed that Ghsp^73*A*sn^ and Gbsp^113*S*er^ mutant proteins did not change the subcellular localization of proteins. Protein interaction analysis showed that GhSP could interact with GhFD1 and Gh14-3-3 in plant cells, but mutant proteins of Ghsp^73*A*sn^ and Gbsp^113*S*er^ could not interact with GhFD1 and therefore it is likely that these mutant SPs cannot form a functional FAC to effect antiflorigen activities, leading to the cluster-bolls and nulliplex-branch phenotypes in cotton (Si et al., 2018). McGarry et al. (2016) found that *GhSP* plays an important role in the regulation of lignin and secondary cell wall synthesis, emphasizing the influence of *SP* on plant stem morphology.

To date, *SP* is considered the most important gene that controls the development of fruit branches and thus, in the regulation of cotton plant architecture. A single amino acid change in Ghsp^73*A*sn^ (D73N) is located in the highly conserved domains D-P-D-x-P, whereas the amino acid change in Gbsp^113*S*er^ (P113S) is located next to the conserved domains G-x-H-R of PEBP proteins (Si et al., 2018). Both domains are located on both sides of anion binding sites (Ahn et al., 2006). Tomato sp^76*L*eu^ (P76L) is also located in the D-P-D-x-P domain, resulting in determinate growth of the terminal buds (Pnueli et al., 1998). It shows that the changes of key amino acids in this domain of plant SP protein may result in the loss of protein function and plant type change, which may be a research direction of targeted gene directional change. The production of further site-directed mutations in SP orthologs will undoubtedly enhance our understanding of their protein-protein interactions with FD and 14-3-3- members in the future.

## The Application of Adjusting the Balance Between Florigen and Antiflorigen: Cotton and Crop Production

During the process of evolution, plants have evolved to coordinate endogenous factors and exogenous environmental stimuli to ensure that flowering occurs at the correct time. After domestication and the application of selection criteria in crops breeding, the flowering time and plant architecture have changed dramatically. Many of the successes in crop breeding for improved yield and architectural traits, can ultimately be related to changes to florigen and antiflorigen, and their inter-play (Eshed and Lippman, 2019).

Genetic evidence from several plant species, including arabidopsis, tomato and rice shows that all flowering pathways eventually converge to the florigen and antiflorigen system, which can be divided into core regulatory components and peripheral regulatory components (Lifschitz et al., 2014; Eshed and Lippman, 2019). Core components play a common role in all flowering plants, such as florigen FT/SFT and antiflorigen TFL1/SP/CEN, and their interacting proteins such as FD, 14-3-3 protein, etc. FT and TFL1 compete with FD proteins to regulate the expression of common target genes of flowering such as *LFY*, *AP1* and others so as to control the determinate or indeterminate growth of meristem (Abe et al., 2005; Wigge et al., 2005; Collani et al., 2019; Romera-Branchat et al., 2020; Zhu et al., 2020). Moreover, the content, proportion and position of expressed FT and TFL1/SP proteins in different tissues and different developmental times accurately regulate the flowering time and architecture of plants (Lifschitz et al., 2014; Moraes et al., 2019). The manipulation of SFT/SP balance in tomato can change the planting pattern and management, and enhance yield (Park et al., 2014b).

The present research showed that in cotton, GhFT/SFT belongs to the florigen component, whereas GhSP exerts the effects of antiflorigen in cotton with a dose effect (McGarry et al., 2016; Liu et al., 2018; Prewitt et al., 2018; Si et al., 2018; Chen et al., 2019). Overexpression of the two cotton *TFL1* (*GhTFL1-1* and *GhTFL1-2*) genes in *A. thaliana* caused a late flowering phenotype, and partially rescued the early flowering phenotype of the *Attfl1-14* mutant. Yeast two-hybrid assay showed that both could interact with an FD homologous protein (Prewitt et al., 2018), suggesting that the functions of *GhTFL1-1* and *GhTFL1-2* are antagonistic to *GhFT*/*SFT* and can be considered components of cotton antiflorigens, but their detailed functions remain unclear. AtTFL1 can also be translocated from its site of synthesis, but in a different way from FT (Conti and Bradley, 2007). *AtTFL1* mRNA expressed in chalazal endosperm is transported to syncytial peripheral endosperm. ChIP-seq analyses have revealed a variety of target genes of AtTFL1-FD (Collani et al., 2019; Goretti et al., 2020). However, one such target is the ABA pathway ABA INSENSITIVE 5 protein which, after AtTFL1-FD binding is involved in seed size regulation (Zhang et al., 2020).

Because no natural mutant related to cotton *TFL1* has been identified and cotton is a recalcitrant plant species for regeneration, an in-depth study of this gene function is practically more difficult. In-depth functional study of cotton *TFL1* gene can better analyze the molecular mechanism of *TFL1*-clade gene regulating cotton plant architecture and establishing the genetic network of branches and plant architecture, which is of great significance in cotton. *SP* mainly regulates axillary meristem and promotes the formation of short fruit branches. At present, the short fruit branches varieties of long-staple cotton and sea-island cotton in production mostly use *sp* mutation, which is very popular because it is suitable for dense planting, convenient management and convenient mechanized harvesting (Si et al., 2018; Chen et al., 2019; Zhan et al., 2021). Because there is no mutant with self-pruning at the top like tomato *sp*, at present, the indeterminate growth of cotton is mainly restrained by spraying DPC and artificial topping with high cost. Genetic analysis of the cotton florigen-antiflorigen system and genetic improvement through gene editing technologies are expected to resolve these cultivation issues in the near future.

## Conclusion

Although breeding and selection practices of cultivated upland and sea-island cotton have led to their day neutrality, the habit of indeterminate growth still persists, whereas a fully determinate growth habit would be preferable for modern cotton cultivation and harvest. At present, the short fruit branch plant architecture represents the most ideal plant type, since it is suitable for dense planting, mechanized harvesting and produces enhanced yield. Scientists have noticed that key factors in hormone signaling pathway play an important role in cotton branch development, such as *BRC1*, *CUC2*, *D14*, and *DREB1B* genes. FT/SFT and SP constitute the components of florigen and antiflorigen in cotton, respectively. The natural allelic variation of *SP* forms the short fruit branch plant type. However, the persistence of some undesirable perennial characteristics in the *sp* mutants used in modern cotton cultivation need to be further studied. Further exploration into the functions of the antiflorigen components, TFL1/SP, and the interactions between FT/SFT, TFL1/SP, and BRC1, as well as analyzing the FAC components and molecular essence of cotton will be the focus of future research. The purpose is to establish the genetic network underlying cotton flowering transition and the regulation of plant architecture. Subsequently, the knowledge gained will be used to finely adjust the balance between florigen and antiflorigen using CRISPR/Cas technology, thus facilitating further genetic improvement in cotton.

## Author Contributions

XH, HL, and BM designed the initial manuscript and contributed reviewing and discussing the manuscript. XH wrote the manuscript. HL conceived the figure. BM checked all the references. All authors contributed to the article and approved the submitted version.

## Conflict of Interest

The authors declare that the research was conducted in the absence of any commercial or financial relationships that could be construed as a potential conflict of interest.

## Publisher’s Note

All claims expressed in this article are solely those of the authors and do not necessarily represent those of their affiliated organizations, or those of the publisher, the editors and the reviewers. Any product that may be evaluated in this article, or claim that may be made by its manufacturer, is not guaranteed or endorsed by the publisher.

## References

[B1] Abe M. Kobayashi Y. Yamamoto S. Daimon Y. Yamaguchi A. Ikeda Y. (2005). FD, a bZIP protein mediating signals from the floral pathway integrator FT at the shoot apex. *Science* 309 1052–1056. 10.1126/science.1115983 16099979

[B2] Ahn J. H. Miller D. Winter V. J. Banfield M. J. Lee J. H. Yoo S. Y. (2006). A divergent external loop confers antagonistic activity on floral regulators FT and TFL1. *EMBO J.* 25 605–614. 10.1038/sj.emboj.7600950 16424903 PMC1383534

[B3] Barton M. K. (2010). Twenty years on: the inner workings of the shoot apical meristem, a developmental dynamo. *Dev. Biol.* 341 95–113. 10.1016/j.ydbio.2009.11.029 19961843

[B4] Bowman J. L. Eshed Y. (2000). Formation and maintenance of the shoot apical meristem. *Trends Plant Sci.* 5 110–115. 10707076 10.1016/s1360-1385(00)01569-7

[B5] Bradley D. Carpenter R. Copsey L. Vincent C. Rothstein S. Coen E. (1996). Control of inflorescence architecture in *Antirrhinum*. *Nature* 379 791–797. 8587601 10.1038/379791a0

[B6] Bradley D. Ratcliffe O. Vincent C. Carpenter R. Coen E. (1997). Inflorescence commitment and architecture in *Arabidopsis*. *Science* 275 80–83. 10.1126/science.275.5296.80 8974397

[B7] Cai D. Liu H. Sang N. Huang X. (2017). Identification and characterization of CONSTANS-like (COL) gene family in upland cotton (*Gossypium hirsutum* L.). *PLoS One* 12:e0179038. 10.1371/journal.pone.0179038 PMC5462432 28591177

[B8] Chen W. Yao J. Li Y. Zhao L. Liu J. Guo Y. (2019). Nulliplex-branch, a TERMINAL FLOWER1 ortholog, controls plant growth habit in cotton. *Theor. Appl. Genet.* 132 97–112. 30288552 10.1007/s00122-018-3197-0

[B9] Chevalier F. Nieminen K. Sánchez-Ferrero J. C. Rodríguez M. L. Chagoyen M. Hardtke C. S. (2014). Strigolactone promotes degradation of DWARF14, an α/β hydrolase essential for strigolactone signaling in *Arabidopsis*. *Plant Cell* 26 1134–1150. 10.1105/tpc.114.122903 24610723 PMC4001374

[B10] Collani S. Neumann M. Yant L. Schmid M. (2019). FT modulates genome-wide DNA-binding of the bZIP transcription factor FD. *Plant Physiol.* 180 367–380. 10.1104/pp.18.01505 30770462 PMC6501114

[B11] Conti L. Bradley D. (2007). TERMINAL FLOWER1 is a mobile signal controlling *Arabidopsis* architecture. *Plant cell* 19 767–778. 10.1105/tpc.106.049767 17369370 PMC1867375

[B12] Diao Y. Zhan J. Zhao Y. Liu L. Liu P. Wei X. (2019). GhTIE1 regulates branching through modulating the transcriptional activity of TCPs in cotton and *Arabidopsis*. *Front. Plant Sci.* 10:1348. 10.3389/fpls.2019.01348 PMC6827420 31719830

[B13] Eshed Y. Lippman Z. B. (2019). Revolutions in agriculture chart a course for targeted breeding of old and new crops. *Science* 366:eaax0025. 10.1126/science.aax0025 31488704

[B14] Gomez-Roldan V. Fermas S. Brewer P. B. Puech-Pagès V. Dun E. A. Pillot J. P. (2008). Strigolactone inhibition of shoot branching. *Nature* 455 189–194. 18690209 10.1038/nature07271

[B15] González-Grandío E. Poza-Carrión C. Sorzano C. O. S. Cubas P. (2013). BRANCHED1 promotes axillary bud dormancy in response to shade in *Arabidopsis*. *Plant Cell* 25 834–850. 10.1105/tpc.112.108480 23524661 PMC3634692

[B16] Gore U. R. (1935). Morphogenic studies of the inflorescence of cotton. *Bot. Gaz.* 97 118–138.

[B17] Goretti D. Silvestre M. Collani S. Langenecker T. Méndez C. Madueño F. (2020). TERMINAL FLOWER1 functions as a mobile transcriptional cofactor in the shoot apical meristem. *Plant Physiol.* 182 2081–2095. 10.1104/pp.19.00867 31996406 PMC7140938

[B18] Grover C. E. Yoo M. J. Lin M. Murphy M. D. Harker D. B. Byers R. L. (2020). Genetic analysis of the transition from wild to domesticated cotton (*Gossypium hirsutum* L.). *G3* 10 731–754. 10.1534/g3.119.400909 31843806 PMC7003101

[B19] Guo D. Li C. Dong R. Li X. Xiao X. Huang X. (2015). Molecular cloning and functional analysis of the FLOWERING LOCUS T (FT) homolog GhFT1 from *Gossypium hirsutum*. *J. Integr. Plant Biol.* 57 522–533. 10.1111/jipb.12316 25429737

[B20] Hu Y. Chen J. Fang L. Zhang Z. Ma W. Niu Y. (2019). Gossypium barbadense and *Gossypium hirsutum* genomes provide insights into the origin and evolution of allotetraploid cotton. *Nat. Genet.* 51 739–748. 30886425 10.1038/s41588-019-0371-5

[B21] Huang C. Nie X. Shen C. You C. Li W. Zhao W. (2017). Population structure and genetic basis of the agronomic traits of upland cotton in China revealed by a genome-wide association study using high-density SNPs. *Plant Biotechnol. J.* 15 1374–1386. 10.1111/pbi.12722 28301713 PMC5633765

[B22] Huang G. Wu Z. Percy R. G. Bai M. Li Y. Frelichowski J. E. (2020). Genome sequence of *Gossypium herbaceum* and genome updates of *Gossypium arboreum* and *Gossypium hirsutum* provide insights into cotton A-genome evolution. *Nat. Genet.* 52 516–524. 10.1038/s41588-020-0607-4 32284579 PMC7203013

[B23] Hubbard L. McSteen P. Doebley J. Hake S. (2002). Expression patterns and mutant phenotype of teosinte branched1 correlate with growth suppression in maize and teosinte. *Genetics* 162 1927–1935. 10.1093/genetics/162.4.1927 12524360 PMC1462370

[B24] Ji G. Liang C. Cai Y. Pan Z. Meng Z. Li Y. (2021). A copy number variant at the HPDA-D12 locus confers compact plant architecture in cotton. *New Phytol.* 229 2091–2103. 10.1111/nph.17059 33129229

[B25] Jin S. Nasim Z. Susila H. Ahn J. H. (2021). Evolution and functional diversification of FLOWERING LOCUS T/TERMINAL FLOWER 1 family genes in plants. *Semin. Cell Dev. Biol.* 109 20–30. 10.1016/j.semcdb.2020.05.007 32507412

[B26] Karlgren A. Gyllenstrand N. Källman T. Sundström J. F. Moore D. Lascoux M. (2011). Evolution of the PEBP gene family in plants: functional diversification in seed plant evolution. *Plant Physiol.* 156 1967–1977. 10.1104/pp.111.176206 21642442 PMC3149940

[B27] Lang A. Chailakhyan M. K. Frolova I. A. (1977). Promotion and inhibition of flower formation in a dayneutral plant in grafts with a short-day plant and a long-day plant. *Proc. Natl. Acad. Sci. U.S.A.* 74 2412–2416. 10.1073/pnas.74.6.2412 16592404 PMC432182

[B28] Li C. Zhang Y. Zhang K. Guo D. Cui B. Wang X. (2015). Promoting flowering, lateral shoot outgrowth, leaf development, and flower abscission in tobacco plants overexpressing cotton FLOWERING LOCUS T (FT)-like gene GhFT1. *Front. Plant Sci.* 6:454. 10.3389/fpls.2015.00454 PMC4469826 26136765

[B29] Lifschitz E. Ayre B. G. Eshed Y. (2014). Florigen and anti-florigen – a systemic mechanism for coordinating growth and termination in flowering plants. *Front. Plant Sci.* 5:465. 10.3389/fpls.2014.00465 PMC4165217 25278944

[B30] Liu D. Teng Z. Kong J. Liu X. Wang W. Zhang X. (2018). Natural variation in a CENTRORADIALIS homolog contributed to cluster fruiting and early maturity in cotton. *BMC Plant Biol.* 18:286. 10.1186/s12870-018-1518-8 PMC6245773 30458710

[B31] Liu H. Huang X. Ma B. Zhang T. Sang N. Zhuo L. (2021). Components and functional diversification of florigen activation complexes in cotton. *Plant Cell Physiol.* 62 1542–1555. 10.1093/pcp/pcab107 34245289

[B32] McGarry R. C. Ayre B. G. (2012a). Manipulation plant architecture with members of the CETS gene family. *Plant Sci.* 188 71–81. 10.1016/j.plantsci.2012.03.002 22525246

[B33] McGarry R. C. Ayre B. G. (2012b). Geminivirus-mediated delivery of florigen promotes determinate growth in aerial organs and uncouples flowering from photoperiod in cotton. *PLoS One* 7:e36746. 10.1371/journal.pone.0036746 PMC3352926 22615805

[B34] McGarry R. C. Ayre B. G. (2021). Cotton architecture: examining the roles of SINGLE FLOWER TRUSS and SELF-PRUNING in regulating growth habits of a woody perennial crop. *Curr. Opin. Plant Biol.* 59:101968. 10.1016/j.pbi.2020.10.001 33418402

[B35] McGarry R. C. Prewitt S. Ayre B. G. (2013). Overexpression of FT in cotton affects architecture but not floral organogenesis. *Plant Signal. Behav.* 8:e23602. 10.4161/psb.23602 23333977 PMC7030403

[B36] McGarry R. C. Prewitt S. F. Culpepper S. Eshed Y. Lifschitz E. Ayre B. G. (2016). Monopodial and sympodial branching architecture in cotton is differentially regulated by the *Gossypium hirsutum* SINGLE FLOWER TRUSS and SELF-PRUNING orthologs. *New Phytol.* 212 244–258. 10.1111/nph.14037 27292411

[B37] Moraes T. S. Dornelas M. C. Martinelli A. P. (2019). FT/TFL1: calibrating plant architecture. *Front. Plant Sci.* 10:97. 10.3389/fpls.2019.00097 PMC6381015 30815003

[B38] Ongaro V. Leyser O. (2008). Hormonal control of shoot branching. *J. Exp. Bot.* 59 67–74. 10.1093/jxb/erm134 17728300

[B39] Park S. J. Eshed Y. Lippman Z. B. (2014a). Meristem maturation and inflorescence architecture–lessons from the Solanaceae. *Curr. Opin. Plant Biol.* 17 70–77. 10.1016/j.pbi.2013.11.006 24507497

[B40] Park S. J. Jiang K. Tal L. Yichie Y. Gar O. Zamir D. (2014b). Optimization of crop productivity in tomato using induced mutation in the florigen pathway. *Nat. Genet.* 46 1337–1342. 10.1038/ng.3131 25362485

[B41] Pathak R. S. Singh R. B. (1975). Genetic analysis of the duplicate loci, cluster and short branch in *Gossypium hirsutum* L. *Theor. Appl. Genet.* 46 281–287. 10.1007/BF00281150 24420122

[B42] Pei X. Wang X. Fu G. Chen B. Nazir M. F. Pan Z. (2021). Identification and functional analysis of 9-cis-epoxy carotenoid dioxygenase (NCED) homologs in G. hirsutum. *Int. J. Biol. Macromol.* 182 298–310. 10.1016/j.ijbiomac.2021.03.154 33811933

[B43] Peng J. Richards D. E. Hartley N. M. Murphy G. P. Devos K. M. Flintham J. E. (1999). ‘Green revolution’ genes encode mutant gibberellin response modulators. *Nature* 400 256–261. 10.1038/22307 10421366

[B44] Pnueli L. Carmel-Goren L. Hareven D. Gutfinger T. Alvarez J. Ganal M. (1998). The SELF-PRUNING gene of tomato regulates vegetative to reproductive switching of sympodial meristems and is the ortholog of CEN and TFL1. *Development* 125 1979–1989. 10.1242/dev.125.11.1979 9570763

[B45] Prewitt S. F. Ayre B. G. McGarry R. C. (2018). Cotton CENTRORADIALIS/TERMINAL FLOWER 1/SELF-PRUNING genes functionally diverged to differentially impact plant architecture. *J. Exp. Bot.* 69 403–417. 10.1093/jxb/ery324 30202979 PMC6255698

[B46] Reinhardt D. Kuhlemeier C. (2002). Plant architecture. *EMBO Rep.* 3 846–851. 12223466 10.1093/embo-reports/kvf177 PMC1084230

[B47] Romera-Branchat M. Severing E. Pocard C. Ohr H. Vincent C. Née G. (2020). Functional divergence of the *Arabidopsis* florigen-interacting bZIP transcription factors FD and FDP. *Cell Rep.* 31:107717. 10.1016/j.celrep.2020.107717 PMC7273178 32492426

[B48] Sang N. Cai D. Li C. Sun Y. Huang X. (2019). Characterization and activity analyses of the flowering locus T promoter in *Gossypium Hirsutum*. *Int. J. Mol. Sci.* 20:4769. 10.3390/ijms20194769 31561427 PMC6801411

[B49] Sang N. Liu H. Ma B. Huang X. Zhuo L. Sun Y. (2021). Roles of the 14-3-3 gene family in cotton flowering. *BMC Plant Biol.* 21:162. 10.1186/s12870-021-02923-9 PMC8015177 33789593

[B50] Sasaki A. Ashikari M. Ueguchi-Tanaka M. Itoh H. Nishimura A. Swapan D. (2002). Green revolution: a mutant gibberellin-synthesis gene in rice. *Nature* 416 701–702. 11961544 10.1038/416701a

[B51] Shalit A. Rozman A. Goldshmidt A. Alvarez J. P. Bowman J. L. Eshed Y. (2009). The flowering hormone florigen functions as a general systemic regulator of growth and termination. *Proc. Natl. Acad. Sci. U.S.A.* 106 8392–8397. 10.1073/pnas.0810810106 19416824 PMC2688896

[B52] Si Z. Liu H. Zhu J. Chen J. Wang Q. Fang L. (2018). Mutation of SELF-PRUNING homologs in cotton promotes short-branching plant architecture. *J. Exp. Bot.* 69 2543–2553. 10.1093/jxb/ery093 29547987 PMC5920339

[B53] Song X. L. Zhang T. Z. (2009). Quantitative trait loci controlling plant architectural traits in cotton. *Plant Sci.* 177 317–323.

[B54] Su J. Li L. Zhang C. Wang C. Gu L. Wang H. (2018). Genome-wide association study identified genetic variations and candidate genes for plant architecture component traits in Chinese upland cotton. *Theor. Appl. Genet.* 131 1299–1314. 10.1007/s00122-018-3079-5 29497767

[B55] Sun Q. Xie Y. Li H. Liu J. Geng R. Wang P. (2021). Cotton GhBRC1 regulates branching, flowering, and growth by integrating multiple hormone pathways. *Crop J.* 10 75–87.

[B56] Susila H. Jurić S. Liu L. Gawarecka K. Chung K. S. Jin S. (2021). Florigen sequestration in cellular membranes modulates temperature-responsive flowering. *Science* 373 1137–1142. 10.1126/science.abh4054 34516842

[B57] Tao Q. Guo D. Wei B. Zhang F. Pang C. Jiang H. (2013). The TIE1 transcriptional repressor links TCP transcription factors with TOPLESS/TOPLESS-RELATED corepressors and modulates leaf development in *Arabidopsis*. *Plant Cell* 25 421–437. 10.1105/tpc.113.109223 23444332 PMC3608769

[B58] Taoka K. I. Ohki I. Tsuji H. Furuita K. Hayashi K. Yanase T. (2011). 14-3-3 proteins act as intracellular receptors for rice Hd3a florigen. *Nature* 476 332–335. 10.1038/nature10272 21804566

[B59] Tian Z. Wang X. Lee R. Li Y. Specht J. E. Nelson R. L. (2010). Artificial selection for determinate growth habit in soybean. *Proc. Natl. Acad. Sci. U.S.A.* 107 8563–8568. 10.1073/pnas.1000088107 20421496 PMC2889302

[B60] Wang B. Smith S. M. Li J. (2018). Genetic regulation of shoot architecture. *Annu. Rev. Plant Biol.* 69 437–468. 29553800 10.1146/annurev-arplant-042817-040422

[B61] Wang C. Ma Qi Xie X. Zhang X. Yang D. Su J. (2022). Identification of favorable haplotypes/alleles and candidate genes for three plant architecture-related traits via a restricted two-stage multilocus genome-wide association study in upland cotton. *Crop. Prod.* 177:114458.

[B62] Wang P. Zhang S. Qiao J. Sun Q. Shi Q. Cai C. (2019). Functional analysis of the GbDWARF14 gene associated with branching development in cotton. *Peer J.* 7:e6901. 10.7717/peerj.6901 31143538 PMC6524629

[B63] Wen T. W. Dai B. S. Wang T. Liu X. X. You C. Y. Lin Z. X. (2019). Genetic variations in plant architecture traits in cotton (*Gossypium hirsutum*) revealed by a genome wide association study. *Crop J.* 7 85–92.

[B64] Wigge P. A. Kim M. C. Jaeger K. E. Busch W. Schmid M. Lohmann J. U. (2005). Integration of spatial and temporal information during floral induction in *Arabidopsis*. *Science* 309 1056–1059. 10.1126/science.1114358 16099980

[B65] Wu H. Ren Z. Zheng L. Guo M. Yang J. Hou L. (2021). The bHLH transcription factor GhPAS1 mediates BR signaling to regulate plant development and architecture in cotton. *Crop J.* 9 1049–1059.

[B66] Yang Z. Zhang C. Yang X. Liu K. Wu Z. Zhang X. (2014). PAG1, a cotton brassinosteroid catabolism gene, modulates fiber elongation. *New Phytol.* 203 437–448. 10.1111/nph.12824 24786710

[B67] Yao R. Ming Z. Yan L. Li S. Wang F. Ma S. (2016). DWARF14 is a non-canonical hormone receptor for strigolactone. *Nature* 536 469–473. 10.1038/nature19073 27479325

[B68] Zhan J. Chu Y. Wang Y. Diao Y. Zhao Y. Liu L. (2021). The miR164-GhCUC2-GhBRC1 module regulates plant architecture through abscisic acid in cotton. *Plant Biotechnol. J.* 19 1839–1851. 10.1111/pbi.13599 33960609 PMC8428825

[B69] Zhang B. Li C. Li Y. Yu H. (2020). Mobile TERMINAL FLOWER1 determines seed size in *Arabidopsis*. *Nat. Plants* 6 1146–1157. 10.1038/s41477-020-0749-5 32839516

[B70] Zhu Y. Klasfeld S. Jeong C. W. Jin R. Goto K. Yamaguchi N. (2020). TERMINAL FLOWER 1-FD complex target genes and competition with FLOWERING LOCUS T. *Nat. Commun.* 11:5118. 10.1038/s41467-020-18782-1 33046692 PMC7550357

